# Self-transformation and structural reconfiguration in coacervate-based protocells†
†Electronic supplementary information (ESI) available: Turbidity plots, DLS data, rheology data, coacervate size distribution and TEM images, confocal and epifluorescence microscopy images and movies. See DOI: 10.1039/c6sc00205f


**DOI:** 10.1039/c6sc00205f

**Published:** 2016-05-25

**Authors:** Ravinash Krishna Kumar, Robert L. Harniman, Avinash J. Patil, Stephen Mann

**Affiliations:** a Centre for Protolife Research and Centre for Organized Matter , School of Chemistry , University of Bristol , Bristol , BS8 1TS , UK . Email: s.mann@bristol.ac.uk

## Abstract

A functionalized small-molecule dipeptide capable of structural adaptation is used to prepare coacervate-based protocells that exhibit a pH-triggered process of self-transformation and structural reconfiguration. Polymer-dipeptide coacervate micro-droplets are prepared at pH 8.5 from aqueous mixtures of poly(diallyldimethylammonium chloride) and deprotonated *N*-(fluorenyl-9-methoxycarbonyl)-d-Ala-d-Ala, and transform into discrete aster-like micro-architectures by controlled lowering of the pH to 4.5. Reconfiguration of the micro-droplets results in entanglement and formation of an interpenetrating fibrous network that subsequently develops into a polymer-containing dipeptide hydrogel. Our results provide a step towards the assembly of synthetic protocells exhibiting rudimentary aspects of metamorphosis, and should offer a new approach to the design and construction of soft reconfigurable chemical micro-ensembles.

## Abstract 2

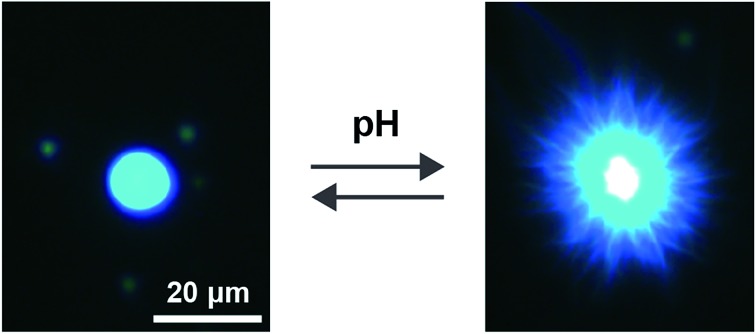
We highlight a new approach for the design and construction of re-configurable soft colloidal scale objects (protocells) based on the pH-induced transition of dipeptide-containing coacervate micro-droplets into discrete aster-like micro-architectures.

## Introduction

The design and construction of reconfigurable soft matter is an essential paradigm for understanding the dynamic and adaptive behaviour of living systems. Moreover, reconfigurable systems that sense and respond structurally and functionally to external stimuli are important for engineering actuated materials and dynamic polymer systems,^1^ developing novel strategies in regenerative medicine^2^,^3^ and extending the emerging area of protocell research.^4^,^5^ We recently demonstrated that coacervate micro-droplets prepared by electrostatically induced complexation of counter-charged polyelectrolytes or polyelectrolyte/small molecule aqueous mixtures can be developed as membrane-free, molecularly crowded protocells,^6^ and herein we explore the possibility of exploiting these organized micro-ensembles as novel types of soft reconfigurable systems.

Coacervate micro-droplets are produced by liquid–liquid phase separation and exhibit a range of biomimetic properties such as selective molecular uptake,^7^,^8^ micro-compartmentalized nanoparticle^9^ or enzyme catalysis,^10^*in vitro* gene expression,^11^,^12^ and templating of lipid membrane multilayer assembly.^13^ Although recent studies have used auxiliary components such as inorganic nanoparticles,^14^ inorganic polyanionic clusters,^15^ covalent crosslinking,^16^,^17^ and hydrogels^18^,^19^ to produce higher-order coacervate-based micro-architectures, the use of coacervate micro-droplets as an intrinsic, structurally reconfigurable micro-compartmentalized phase has been rarely exploited.^20^ Coacervates based on polymer/small molecule (monomer) complexation show considerable promise as reconfigurable protocells because the relative weakness of the electrostatic interactions increases the scope to structurally and compositionally manipulate the micro-droplets by environmental triggers such as changes in salt concentration, pH and temperature.

**Fig. 1:**
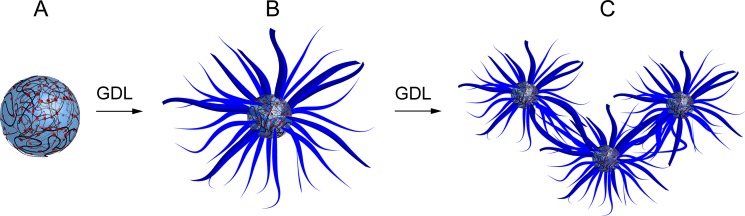
Scheme showing metamorphosis of coacervate-based protocells into entangled hydrogel networks. (A) Polymer-dipeptide (PDDA/FMOC-AA) coacervate micro-droplets prepared at pH 8.5; (B) surface-specific reconfiguration to produce aster-like core/shell micro-architectures at pH 4.5; and (C) entanglement of FMOC-AA nanofibres to produce a macroscopic polymer-containing dipeptide hydrogel. The transformation is induced by slow hydrolysis of glucono-δ-lactone (GDL) in the continuous phase of the coacervate dispersion.

In this paper, we introduce a new strategy for the preparation of coacervate micro-droplets capable of undergoing a pH-triggered process of self-transformation and structural reconfiguration. For this, we design and prepare a novel polymer-dipeptide coacervate based on the electrostatically mediated complexation of poly(diallyldimethylammonium chloride) (PDDA) and deprotonated *N*-(fluorenyl-9-methoxycarbonyl)-d-Ala-d-Ala (FMOC-AA). In the absence of PDDA, FMOC-AA exists as a monomer in aqueous solution at pH 8, but readily self-assembles into a hydrogel of supramolecular nanofibres when the pH is lowered below the p*K*_a_ of the carboxylic acid group.^21^ Significantly, we exploit this reversible transformation to prepare coacervate micro-droplets that are capable of a pH-induced structural adaptation from a molecularly crowded liquid phase (pH 8.5) to a nanofibrous hydrogel network (pH 4.5) (Fig. 1). Our results provide a step towards the assembly of biomimetic micro-droplets exhibiting rudimentary aspects of metamorphosis, and should offer a new approach to the design and construction of soft, reconfigurable micro-ensembles for applications in diverse areas such as sensing, biomedical devices and cell/protocell integration.

## Results and discussion

Polymer-dipeptide coacervates were prepared from mixtures of PDDA (average *M*_w_ = 100 kDa) and FMOC-AA at a range of final monomer concentrations and PDDA/FMOC-AA monomer molar ratios (Experimental methods). In general, turbid suspensions of micro-droplets were obtained at room temperature and pH 8.5 for mixtures containing 20 mM PDDA monomer units and FMOC-AA concentrations greater than 9 mM (ESI, Fig. S1†). The mean size of the droplets after mixing for 1 minute ranged between 200 nm and 5 μm depending on the PDDA monomer/FMOC-AA molar ratio (ESI, Fig. S2, Table S1†), and was attributed to changes in the rate of coalescence associated with variations in the surface charge of the micro-droplets. Thus, samples prepared at approximately equimolar concentrations or in the presence of excess FMOC-AA were susceptible to coalescence and sedimentation into a bulk phase within 5 to 10 minutes, whilst positively charged PDDA-rich droplets with a typical zeta potential value of +35 mV and mean size of *ca.* 240 nm remained in suspension even when centrifuged at 16 100 g for 5 minutes. Under close to charge neutral conditions, the concentration of dipeptide within the continuous aqueous phase was *ca.* 4 mM (ESI, Fig. S3†), which indicated that 80% of the FMOC-AA was complexed within the coacervate medium to give a local concentration of around 450 mM.

**Fig. 2:**
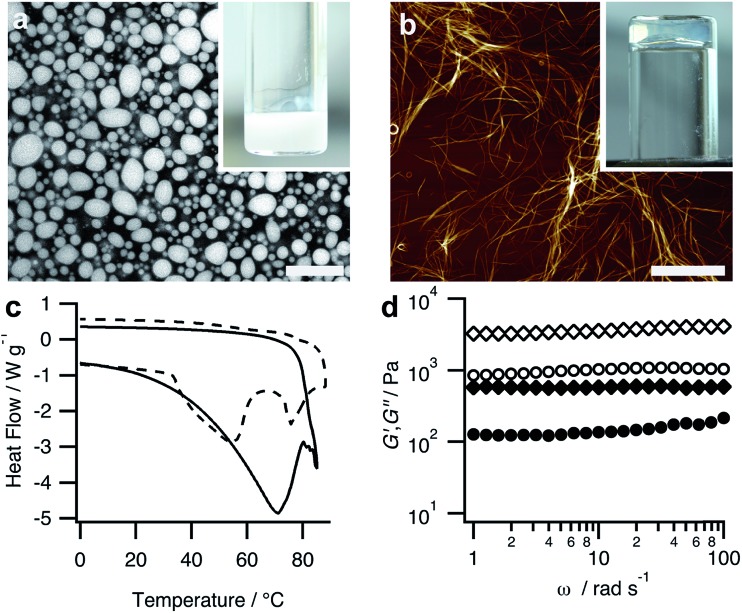
(a) Confocal fluorescence microscopy image showing Hoechst 33258-stained PDDA/FMOC-AA coacervate micro-droplets prepared at a PDDA : FMOC-AA monomer molar ratio = 1 : 1; scale bar = 18 μm. Inset shows sample tube containing bulk coacervate in the form of a turbid dispersion. (b) AFM image showing bundled nanofilaments of protonated FMOC-AA produced after addition of GDL to a PDDA/FMOC-AA coacervate and ageing at room temperature for 1 day to produce a bulk hydrogel; scale bar = 10 μm. Inset shows inverted sample tube with a coacervate-derived PDDA-containing FMOC-AA hydrogel. (c) DSC profiles for hydrogels produced by pH-mediated transformation in PDDA/FMOC-AA coacervate media (solid line) or bulk solutions of FMOC-AA (dashed line). Gel-to-sol melting transitions are observed at 72 and 55 °C, respectively. (d) Frequency sweeps in the linear viscoelastic region showing plots of storage (elastic, *G*′, diamond symbols) and loss (viscous, *G*′′, circles) moduli against frequency for FMOC-AA hydrogels prepared by pH-mediated transformations in PDDA/FMOC-AA coacervate media (closed symbols) or bulk solutions of FMOC-AA (open symbols).

Transformation of the PDDA/FMOC-AA coacervate into a dipeptide hydrogel was achieved by addition of small aliquots of glucono-δ-lactone (GDL, final concentration *ca.* 20 mM) and leaving the unstirred mixture to age at room temperature (ESI,† Experimental methods). Typically, the polymer-dipeptide micro-droplets transformed into a self-supporting PDDA-containing matrix of bundled supramolecular nanofilaments within 24 h (Fig. 2a and b). The self-structuring process was associated with a slow decrease in pH to a value of 4.5 over a period of 16 h (ESI, Fig. S4†). Plots of the time-dependent decrease in pH were consistent with previously reported profiles for FMOC-dipeptide self-assembly,^22^,^23^ and showed a short intermediate period in which the pH slightly increased or remained constant due to buffering of FMOC-AA *via* differences in the p*K*_a_ values of the monomeric and self-assembled forms of the dipeptide.^24^ This region of pH invariance, which has been correlated with the onset of FMOC-peptide self-assembly,^23^ was delayed in the coacervate medium (*t*_lag_ = 9.6 ± 2.7 min) compared with self-assembly of FMOC-AA in bulk solution under the same conditions (*t*_lag_ = 3.9 ± 1.8 min). It seems feasible that electrostatic interactions with the cationic PDDA chains, or the high level of molecular crowding, or both, were responsible for reducing the rate of FMOC-AA deprotonation and hence curtailing the onset of nanofilament nucleation. Given the non-covalent nature of the interactions responsible for both liquid–liquid micro-phase separation and nanofilament self-assembly, it was possible to transition reversibly between the coacervate and hydrogel phases by appropriate control of the solution pH to regulate the deprotonation/protonation state of FMOC-AA using combinations of GDL/dilute NaOH or gaseous CO_2_/NH_3_ (ESI, Fig. S5†).

We used a range of microscopic and physical methods to assess the properties of the coacervate-derived hydrogels compared with hydrogels prepared by acidification of 20 mM FMOC-AA bulk solutions in the absence of PDDA (ESI,† Experimental methods). The coacervate-derived and FMOC-AA control hydrogels were visually similar, and consisted of entangled dipeptide nanofibres comprising laterally bundled protofilaments with mean widths of 3.8 ± 0.9 nm and 3.5 ± 0.9 nm, respectively (ESI, Fig. S6†). In both cases, circular dichroism (CD) spectra of the hydrogels showed bands at 217 nm (n–π*) and 231–303 nm (π–π*) (ESI, Fig. S7†), indicating a similar superhelical arrangement of alanine and fluorenyl residues within the dipeptide nanofilaments.^21^ Differential scanning calorimetry (DSC) profiles showed broad melting transitions at around 72 or 55 °C for hydrogels prepared by coacervate-derived transformation or in bulk solution, respectively (Fig. 2c). The increased gel-to-sol transition temperature of the coacervate-derived FMOC-AA hydrogel was attributed to increased stabilization of the dipeptide nanobundles due to favourable interactions with the PDDA chains. On the other hand, the presence of PDDA reduced the mechanical strength of the hydrogel to shear-induced strain compared with analogous materials prepared in bulk solution. In particular, frequency sweeps in the linear viscoelastic region gave elastic moduli (*G*′) values at 10 rad s^–1^ of approximately 510 and 4300 Pa, and corresponding loss factors (tan *δ*) of 0.288 and 0.277 for the coacervate-derived and control hydrogels, respectively (Fig. 2d), indicating that the former was less solid-like.^25^ However, the deformation properties of both hydrogels were similar, showing crossover points from the gel to liquid state at similar strain values (ESI, Fig. S8†).

**Fig. 3:**
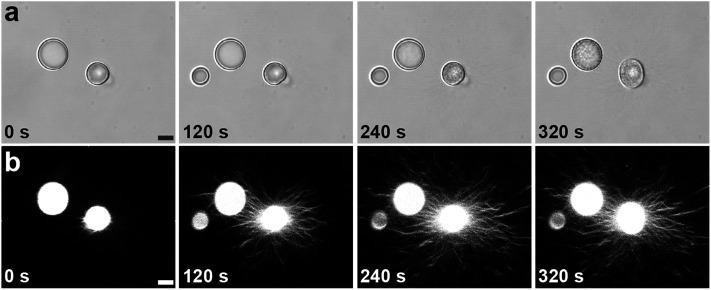
Time-dependent series of images recorded by (a) transmitted light microscopy or (b) confocal microscopy after addition of GDL to a suspension of PDDA/FMOC-AA coacervate micro-droplets. Changes in surface texture (a), and the onset of fibrous outgrowths of dipeptide nanofibres (b) are observed within a period of 320 s. Scale bars = 8 μm.

The above results demonstrate that by using a structurally adaptive pH-responsive functionalized dipeptide it is possible to prepare coacervates capable of undergoing triggered processes of self-transformation and reconfiguration. To further elucidate these processes, we used epifluorescence and confocal fluorescence microscopy to monitor the time-dependent structural and morphological changes associated with individual PDDA/FMOC-AA micro-droplets whilst undergoing transformation. The droplets (1–30 μm in size) were prepared at a PDDA : FMOC-AA monomer molar ratio of 1 : 1, and stained prior to addition of GDL with the peptide nanofibre-binding blue fluorescent dye, Hoechst 33258. Time-dependent optical microscopy images showed a progressive roughening in the texture of the coacervate micro-droplets within a few minutes of GDL addition (Fig. 3a), and corresponding confocal microscopy images showed the emergence of a corona of fibrous outgrowths that emanated in all directions from the surface of individual PDDA/FMOC-AA coacervate droplets (Fig. 3b). This phenomenon of outward fibre growth from coacervate droplets was attributed to the slow hydrolysis rates of GDL and consequential protonation rates of FMOC-AA (ESI, Fig. S4†). If mineral acids were used to lower the pH, such as HCl, aggregates formed (ESI, Fig. S9†) comprising ring-like structures of FMOC-AA around coacervate droplets, which was discerned by the spatial localisation of Hoechst 33258 fluorescence.

**Fig. 4:**
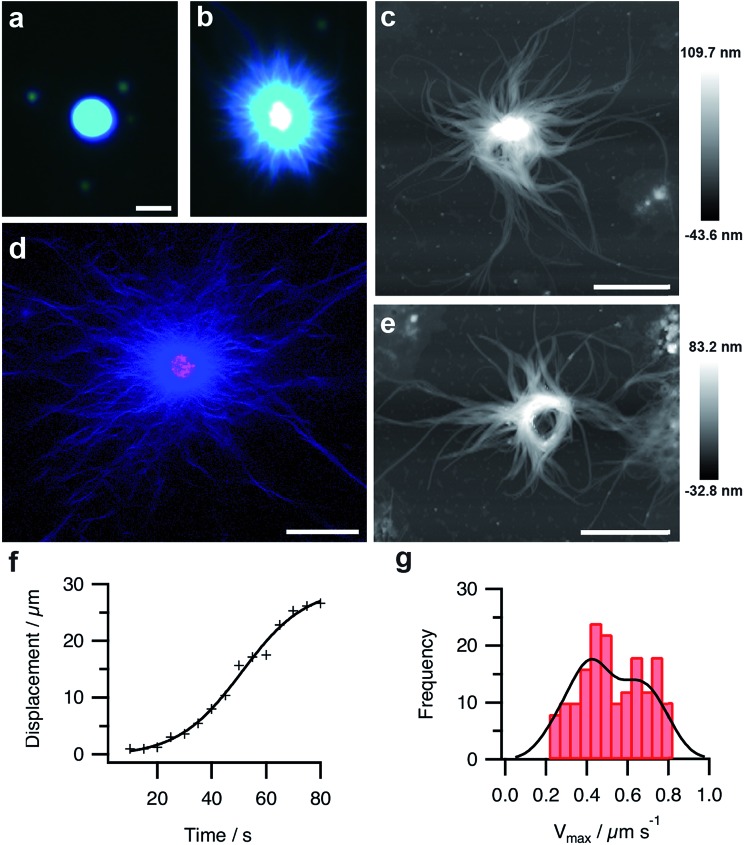
(a and b) Fluorescence microscopy images of a single PDDA/FMOC-AA droplet with sequestered Hoechst 33258 fluorescent dye before (a) and 10 min after (b) addition of GDL. The slow decrease in pH is associated with the emergence of a hairy aster-like shell of dipeptide fibres surrounding the polymer-dipeptide coacervate core; scale bar = 10 μm in both images. (c) AFM height image of a transforming droplet 3 h after addition of GDL showing extended matrix of FMOC-AA outgrowths surrounding a contracted PDDA/FMOC-AA core; scale bar = 2 μm. (d) Two-channel confocal fluorescence microscopy image of an individual aster-like microstructure doped with 1 mol% RITC-labelled PAH. The image shows red (polymer) and blue (dipeptide) fluorescence located specifically in the contracted core and fibrous shell regions, respectively; scale bar = 16 μm. (e) AFM height image of a transforming PDDA/FMOC-AA droplet 3 h after addition of GDL showing the presence of an organized ring-like bundle of dipeptide fibres and absence of a coacervate core; scale bar = 2 μm. (f) Plot showing the growth of a single dipeptide fibre with time during the initial stages of transformation of a coacervate micro-droplet into an aster-like micro-architecture. (g) Histogram showing bimodal distribution in the maximum growth rates (*V*_max_) of individually tracked dipeptide fibres centred at values of 0.4 and 0.7 μm s^–1^.

Fluorescence microscopy images indicated that Hoechst 33258 was homogeneously sequestered into the coacervate droplets prior to addition of GDL (Fig. 4a). On addition of GDL, and after an induction period that depended on the size, number and spacing of the micro-droplets present on the microscope slide, individual coacervate droplets became surrounded by an aster-like corona of densely packed short fibres, which exhibited high intensity blue fluorescence (Fig. 4b). The stained images were consistent with binding of the dye to a supramolecular nanofilamentous assembly of FMOC-AA molecules. Continued growth of the nanofibres produced individual droplets with a contracted core enclosed within an extended mesh of highly elongated FMOC-AA fibres (Fig. 4c). The dipeptide fibres were unbranched, flexible, relatively uniform in width, considerably longer than the coacervate droplet core, and self-limiting with regard to their maximum extension. We attributed the latter to hydrogel formation within the core region, which curtailed nanofibre growth by depleting the concentration of protonated FMOC-AA molecules released at the surface of the transforming coacervate micro-droplets. Significantly, we doped the coacervate mixture with 1 mol% of rhodamine B isothiocyanate (RITC)-labelled poly(allylamine hydrochloride) (PAH), and used confocal fluorescence microscopy to determine the spatial distribution of the cationic polymer within the transforming droplets. The images showed that the polymer was specifically located in the core of the spherulitic structure and not associated strongly with the emanating FMOC-AA fibres (Fig. 4d). In certain circumstances, local alignment of the adjacent dipeptide fibres around the surface of a single coacervate core produced a central ring-like bundle of filaments that was retained in the aged hydrogels (Fig. 4e and ESI, Fig. S10†). Analysis of video images recorded on individual fibres during the initial stages of outgrowth (ESI, Fig. S11†) indicated that the rate of nanofilament assembly followed sigmoidal kinetics (Fig. 4f). A corresponding histogram of the maximum growth rates (*V*_max_) of individually tracked dipeptide fibres showed a bimodal distribution comprising two populations with *V*_max_ values of 0.4 ± 0.16 and 0.7 ± 0.08 μm s^–1^ (Fig. 4g), which were attributed to the growth of bundled co-aligned fibres and single filaments, respectively.

**Fig. 5:**
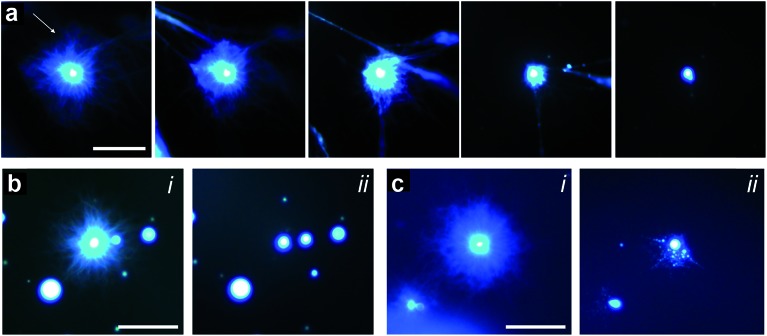
(a) Epifluorescence images showing reconfiguration of an aster-like structure into a single coacervate droplet induced by addition of sodium hydroxide (final concentration = 20 mM). Left to right; time-dependent images recorded sequentially at 10 s intervals after addition of sodium hydroxide. The arrow indicates the direction of the sodium hydroxide gradient; scale bar = 50 μm. (b and c) Fluorescence microscopy images showing two examples of retraction pathways in nanofibre asters subjected to addition of sodium hydroxide; (i) and (ii) represent images recorded before and after addition of sodium hydroxide, respectively. Scale bars = 50 μm.

Given the above observations, we were able to recapitulate formation of the polymer-peptide coacervate micro-droplets by pH-induced transformation of the aster-like dipeptide structures. For this, we added equimolar amounts of sodium hydroxide to counter the addition of GDL, de-protonate FMOC-AA, and reinstall electrostatic interactions between the cationic polymer and functionalized amino acid. Re-formation of the coacervate micro-droplets occurred almost instantaneously upon addition of hydroxide, and was dependent on the hydroxide ion diffusion gradient produced on injection of the alkaline solution (Fig. 5a, ESI, Movie S1†). In general, two main re-assembly pathways were observed involving local retraction of the aster-like structures back into a single coacervate droplet (Fig. 5b), or division into multiple droplets (Fig. 5c, ESI, Movie S2†). We attributed the uncontrollable fission mechanism to turbulent flow associated with the sodium hydroxide gradient, which sheared the nanofibrous structure and induced re-coacervation at multiple sites to produce several daughter droplets. Interestingly, the pH-induced fission process offers a possible route to coacervate micro-droplet division, which we will explore in future work.

**Fig. 6:**
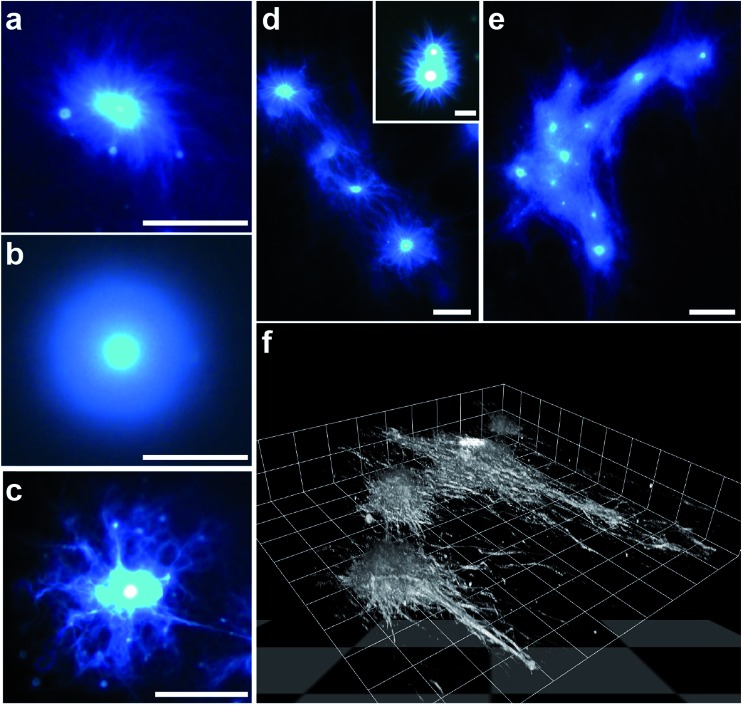
(a–c) Epifluorescence images of transforming PDDA/FMOC-AA coacervate droplets prepared at PDDA (100 kDa) : FMOC-AA molar ratios of 1 : 0.85 (a) and 1 : 0.5 (b), and at a molar ratio of 1 : 1 but with a PDDA molecular weight of 8.5 kDa (c); all scale bars = 50 μm. See text for details. (d and e) Fluorescence images showing formation of dipeptide fibre networks associated with arrays of closely spaced transforming coacervate droplets. Initial stage showing interaction between adjacent FMOC-AA outgrowths (d), and a later phase with a dense interpenetrating fibrous network and coacervate-rich nodes (e); scale bars, (d) 25 μm and 10 μm (inset), and (e) 50 μm. (f) Confocal fluorescence 3D stacked image of a side-view of a self-structuring dipeptide network showing fibres emanating from individual coacervate micro-droplets and spreading across the glass slide. Image was recorded *ca.* 40 min after GDL addition; scale per grid square length = 18 μm. All samples were stained with Hoechst 33258 dye.

The influence of coacervate composition on the self-structuring process was investigated by preparing PDDA/FMOC-AA micro-droplets at lower FMOC-AA concentrations or with a lower molecular weight (8.5 kDa) PDDA polymer. In general, decreasing the monomer molar ratio from 1 : 1 (as described above) to a value of 1 : 0.85 reduced the rate of nucleation and outgrowth of the dipeptide fibrous shell, which in turn produced individual coacervate droplets surrounded by a dense brush-like corona with a spiral texture (Fig. 6a and ESI, Fig. S12a†). Lowering the PDDA/FMOC-AA molar ratio to 1 : 0.5 greatly prolonged the onset of fibre outgrowth to approximately 1 h, and produced a relatively thick, homogeneous shell in which the individual dipeptide fibres could not be resolved by fluorescence microscopy (Fig. 6b and ESI, Fig. S12b†). The slow decomplexation-mediated release of protonated FMOC-AA molecules observed under these conditions suggests that the dipeptide is more strongly bound within the coacervate matrix in the presence of excess PDDA. As a consequence, nucleation and growth of the dipeptide fibres specifically at the droplet surface are less competitive compared with fibre self-assembly arising from released FMOC-AA molecules present in the bulk solution, such that only short, non-distinct fibres are produced in the coronal layer.

Similar experiments were undertaken with polymer/dipeptide coacervate droplets prepared at a molar ratio of 1 : 1 but using a PDDA polymer with an average molecular weight of 8.5 kDa in place of the 100 kDa polymer employed in the experiments described above. Use of the shorter chain polycation was expected to destabilize the coacervate matrix by reducing the attractive interactions between the polymer and FMOC-AA components. As a consequence, although nanofibre aster-like microstructures were readily produced under these conditions, the emanating dipeptide fibrous bundles were thinner, less well-defined, and more branched than those observed in the presence of the 100 kDa polymer (Fig. 6c and ESI, Fig. S12c†). The change in morphology was consistent with the observed faster kinetics of droplet transformation, and indicated that self-assembly of the dipeptide fibres specifically on the droplet surface was highly competitive when compared with nucleation and growth in free solution.

Given the highly anisotropic nature of the dipeptide outgrowths associated with individually transforming coacervate droplets, we also undertook preliminary studies to determine whether 2D assemblies of PDDA/FMOC-AA (1 : 1) micro-droplets could be exploited for the generation of a self-structured environment of interpenetrating fibre networks. For this, we mounted samples of closely spaced coacervate droplets onto PEG-functionalised glass capillary slides and added GDL to initiate droplet transformation. Depending on the separation distance between droplets, the fibre outgrowths became intertwined within 30–40 min with neighbouring aster-like microstructures to form an interconnected fibrous matrix. The degree of entanglement appeared to be controlled by the size of initial droplets, number density within a localized area, and inter-droplet distances (Fig. 6d and e). 3D stacked images obtained from confocal fluorescence microscopy showed a micro-ensemble of interconnecting dipeptide fibres emanating from a series of coacervate nodal points and propagating principally along the surface of the supporting glass substrate (Fig. 6f and ESI Fig. S13†).

## Conclusion

In conclusion, our results indicate that rudimentary aspects of metamorphosis can be integrated into coacervate-based protocells by using a pH-responsive, structure-adaptive dipeptide as a building block of the phase-separated mixture. Transformation of the spherical PDDA/FMOC-AA micro-droplets into discrete aster-like micro-architectures is dependent on a pH diffusion gradient generated by the controlled release of H^+^ ions accompanying the slow hydrolysis of GDL in the continuous phase. Protonation of FMOC-AA results in decomplexation of the coacervate matrix and concomitant self-assembly and outgrowth of a densely packed corona of dipeptide nanofibres specifically on the surface of individual micro-droplets. In contrast, experiments in which GDL was substituted for a stronger acid (HCl) produced discrete hydrogelled coacervate particles with spherical morphology and non-hairy surface texture. Under these conditions, rapid protonation of the dipeptide molecules results in spontaneous nucleation and assembly of the FMOC-AA nanofibres throughout the coacervate micro-droplets rather than outgrowth at the droplet/water interface along an established pH diffusion gradient, indicating that formation of the aster-like structures is under kinetic control. Significantly, our studies indicate that reconfiguration of the protocells results in entanglement of the aster-like microstructures and subsequent formation of an interpenetrating reversible fibrous network that slowly and reversibly transforms into a polymer-containing dipeptide hydrogel. In general, our results suggest that the ability to integrate primitive aspects of structural and morphological transformations in coacervate micro-droplets offers a step towards the design and construction of soft, reconfigurable chemical micro-ensembles, and provides new opportunities for applications in areas such as environmental sensing, biomedical and bio-inspired materials engineering, and storage/release technologies.

## Experimental

### Preparation and transformation of polymer-dipeptide coacervate micro-droplets

Poly(diallyldimethylammonium chloride) (PDDA) with a molecular weight of 100 kDa (*ca.* 620 monomer units, monomer = 161.7 g mol^–1^) or 8.5 kDa (*ca.* 50 monomers) was dissolved in water (pH = 8–9) at a monomer concentration of 40 mM. Coacervates were prepared by addition of 100 μL of aqueous *N*-(fluorenyl-9-methoxycarbonyl)-d-Ala-d-Ala (FMOC-AA, 20–40 mM, Bachem) to a 100 μL aqueous solution of PDDA (20–40 mM in monomer) at a final pH of 8.5. The final PDDA/FMOC-AA monomer molar ratios were 1 : 1, 1 : 0.85 and 1 : 0.5 (100 kDa PDDA), or 1 : 1 (8.5 kDa PDDA). In each case, the turbid suspension of liquid micro-droplets was centrifuged at 16 100 g for 5 min to produce a bulk continuous coacervate phase and supernatant. The samples were then gently agitated with a plastic pipette to re-suspend the bulk coacervate into micro-droplets that were larger than the primary droplets. Typically, droplet sizes of *ca.* 3–4 μm were produced, along with a significant proportion of droplets greater than 10 μm (ESI, Fig. S14†). The latter were routinely imaged using a range of microscopy techniques.

The concentration of FMOC-AA in the measured volume of the supernatant phase, produced by centrifugation of the PDDA/FMOC-AA coacervates into a bulk phase (pelleted coacervate phase) was determined by UV-vis spectroscopy (FMOC-AA extinction coefficient at 265 nm = 16 553 M^–1^ cm^–1^). The difference between the total concentration used in the preparation, and concentration determined in the supernatant was employed to determine the FMOC-AA concentration in the measured volume of the bulk coacervate phase.

Transformation of the PDDA/FMOC-AA micro-droplets into dipeptide nanofilaments and subsequent extension into a hydrogel network was initiated by addition of 1–2 μL of a 2 M glucono-δ-lactone (GDL; final concentration *ca.* 20 mM) solution to 200 μL of a coacervate suspension to reduce the pH from 8.5 to around 4. Typically, the initial stages of transformation occurred within a few hours after adding GDL. After leaving these samples to age for 1 day at room temperature, a self-supporting hydrogel was produced. Alternatively, hydrogelation of the polymer-dipeptide coacervates was achieved by addition of CO_2(g)_ above a freshly prepared suspension of 1 mL PDDA (100 kDa)/FMOC-AA (1 : 1) coacervate solution at a rate of 0.3 L min^–1^. The dispersion typically gelled within 2 hours. Reversible re-assembly of the coacervate droplets was achieved by room temperature addition of sodium hydroxide (20 mM, final pH *ca.* = 8.5) to the network of peptide nanofilaments, or alternatively, by addition of NH_3_ vapour from a 35 wt% aqueous ammonia solution placed above the hydrogel for 5 minutes (final pH of 8.8).

### Imaging studies

Transformation of the polymer-dipeptide micro-droplets after addition of aqueous GDL was studied by epifluorescence or confocal microscopy. After mixing, the samples were immediately mounted onto polyethylene glycol (PEG)-functionalised capillary slides and imaged using a Leica DMI3000B inverted microscope with equipped 40× or 20× lenses, or a Leica SP8 AOBS confocal laser scanning microscope equipped with a glycerol immersion 63× lens and an automated shutter control capable of imaging every 5 seconds. Samples were loaded onto PEG-functionalized capillary slides to avoid wetting of the coacervate droplets. Spatial localization of PDDA during the transformation process was determined by doping the cationic polymer with 1 mol% rhodamine B isothiocyanate (RITC)-labelled poly(allylamine hydrochloride) (RITC-PAH; PDDA : RITC-PAH = 50 : 1). Formation of the FMOC-AA nanofilaments was tracked by addition of 20 μM of the peptide fibre-binding dye Hoechst 33258 to the PDDA/FMOC-AA coacervate suspension. Video images were recorded from a time series of confocal microscopy images using the Manual Tracking plugin in Fiji. The scalar distance from the base to the tip of the individually growing nanofilaments was determined at 5 s time intervals. The maximum growth rate (*V*_max_) was calculated from individual tracks using a purpose written script in MATLAB®. The script fitted a sigmoid function to the tip-to-base displacement over time of an individually tracked filament and calculated the gradient at the inflection point to determine *V*_max_. Approximately 170 filaments were tracked from 20 different coacervate droplets. A kernel density estimate was used to determine the probability density function of the collected growth rates. Log normal functions were fitted to the kernel density estimate to calculate the average growth rates of different populations.

The reversibility of transformed nanofibrous asters was monitored by epifluorescence microscopy after addition of sodium hydroxide to PDDA (100 kDa)/FMOC-AA (1 : 1) systems after 20–30 minutes of GDL addition. Typically 20 mM of sodium hydroxide was added to solutions in capillary slides creating diffusion of sodium hydroxide from the top to the bottom of the sample. As the sodium hydroxide gradient approached aster-like structures, reassembly occurred back to coacervate droplets.

### Dipeptide solution preparation and hydrogel formation in bulk solution

Typically, 200 μL of 1 M sodium hydroxide was added in 1–10 μL aliquots to a 4.8 mL aqueous suspension of *N*-(fluorenyl-9-methoxycarbonyl)-d-Ala-d-Ala (FMOC-AA) to produce an aqueous solution of the dipeptide (40 mM, final pH of 8.5). Dissolution of FMOC-AA was facilitated by sonication of the suspension after addition of each NaOH aliquot using an Ultrawave Q-Series ultrasonication bath. The pH was kept below pH 9 during addition of NaOH to prevent disassociation of the fluorenyl group. The FMOC-AA solution was then passed through a 200 μm filter, and used within three days of preparation. FMOC-AA hydrogels were prepared by addition of glucono-δ-lactone (GDL, final concentration, 20 mM) to a 20 mM FMOC-AA aqueous solution at pH 8.5, and left to age for 24 hours before analysis.

### Preparation of rhodamine B isothiocyanate-tagged poly(allylamine hydrochloride)

A 10 mg mL^–1^ solution of poly(allylamine hydrochloride) (PAH) (15 kDa) was prepared in 100 mM of EPPS buffer (4-(2-hydroxyethyl)-1-piperazinepropanesulfonic acid) at pH 9.5. A volume of 2.83 mL of a solution of rhodamine B isothiocyanate (RITC) dissolved in DMSO (1 mg mL^–1^) was added dropwise to 10 mL of the PAH solution. The reaction was incubated overnight in the dark and under constant stirring to produce RITC-labelled PAH. Removal of unreacted RITC and buffer was achieved by dialysis (molecular weight cut-off of 7000 Da) against Milli-Q water over two days with regular changes of the water. The RITC-PAH solution was then lyophilized and stored in the dark prior to use. UV-vis spectroscopy (RITC extinction coefficient, *ε*_(559 nm)_ = 62 100 M^–1^ cm^–1^) was used to determine the RITC : PAH-monomer molar ratio. Typically, the reaction produced a dye-labelled polymer with a RITC : PAH-monomer molar ratio of 1 : 60.

### Determination of critical coacervation concentration

The critical coacervation concentration (CCC) associated with formation of the PDDA/FMOC-AA coacervate was determined by monitoring the increase in turbidity of an aqueous solution of PDDA (20 mM, 100 kDa, pH 8.5) on addition of 0.5 mM increments of aqueous FMOC-AA. The turbidity was measured using a Perkin Elmer Lambda 25 UV-vis spectrometer and by monitoring the changes in absorbance (*A*) at a fixed wavelength (*λ* = 500 nm). Turbidity values were calculated from (100 – % transmission (*T*)) where % *T* = 100 × 10^–*A*^. The CCC was determined at the point of rapid increase in turbidity associated with formation of the coacervate droplets. The concentration of FMOC-AA in the supernatant phase produced by centrifugation of the PDDA/FMOC-AA coacervates was determined by UV-vis spectroscopy (FMOC-AA extinction coefficient of *ε*_(265 nm)_ = 16 553 M^–1^ cm^–1^). The extinction coefficient was determined by measuring known concentrations of FMOC-AA to determine a linear fit.

### General methods

Dynamic light scattering (DLS) and zeta potential measurements were performed using a Malvern Zetasizer Nano-ZS equipped with an internal Peltier stage. Hydrodynamic diameters and zeta potentials associated with coacervation were determined 1 min after mixing of the PDDA and FMOC-AA solutions at pH 8.5. Circular dichroism (CD) spectroscopy measurements on dipeptide hydrogels prepared from aqueous FMOC-AA solutions or PDDA/FMOC-AA coacervates were undertaken at 25 °C using a JASCO J-815 spectropolarimeter fitted with a Peltier stage. The hydrogels were placed between two quartz plates prior to CD analysis. Transmission Electron Microscopy (TEM) was undertaken on a Jeol TEM 2010 using a LaB_6_ filament at 120 keV in bright field mode. Imaging of FMOC-AA nanofilaments was performed by diluting 10 μL of a hydrogel into 100 μL of deionised water followed by mounting 5 μL of the dispersion onto a carbon-coated copper grid and left to dry at room temperature. Negative staining of the peptide nanofilaments was achieved by mounting 5 μL of a 1 wt% solution of uranyl acetate onto the TEM grid and then drying with filter paper after 5 minutes. AFM studies were performed by depositing samples onto freshly cleaved muscovite mica and drying with compressed nitrogen after set time intervals. AFM imaging was conducted using a Multi-mode VIII microscope utilising Peakforce control (Bruker, USA) in ambient conditions. Cantilevers with spring constant 0.4 N m^–1^ (Bruker, SCANASYST-AIR-HR) were used with the Bruker high-speed scan head to achieve high resolution imaging of regions up to 100 μm.

## Supplementary Material

Supplementary movie

Supplementary movie

Supplementary information
