# Supplementary material for: Self-transformation and structural reconfiguration in coacervate-based protocells
Source: Chem Sci. 2016 May 25;7(9):5879–87. doi: 10.1039/c6sc00205f (PMC6024302; doi:10.1039/c6sc00205f)
Supplement: Supplementary file 3 [file SC-007-C6SC00205F-s003.pdf]

Electronic Supplementary Material (ESI) for Chemical Science.
This journal is © The Royal Society of Chemistry 2016

Self-transformation and structural reconfiguration in coacervate-based protocells

Ravinash Krishna Kumar, Robert L. Harniman, Avinash J. Patil and Stephen Mann

Centre for Protolife Research, Centre for Organized Matter, School of Chemistry, University of Bristol, Bristol, BS8 1TS (UK).

Supporting Information: Figures

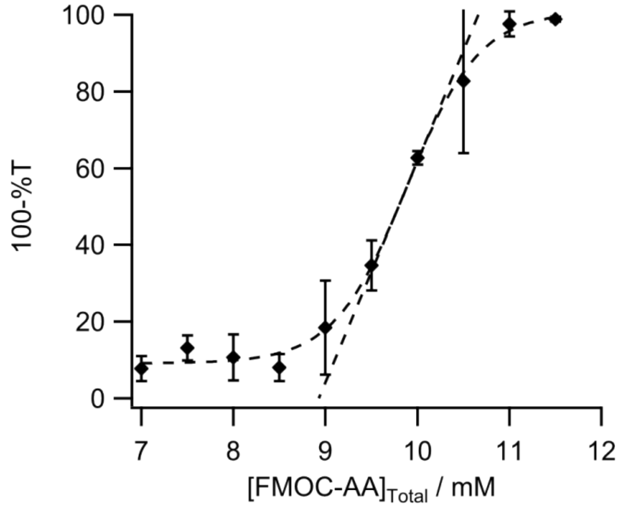

Figure S1. Plot of turbidity (100 - %T; T = transmission) against total concentrations of FMOC-AA ([FMOC-AA]$_{Total}$) added to an aqueous solution of PDDA (100 kDa, 20 mM). The minimum concentration of FMOC-AA required for coacervation (critical coacervation concentration) under these conditions was *ca.* 9 mM.

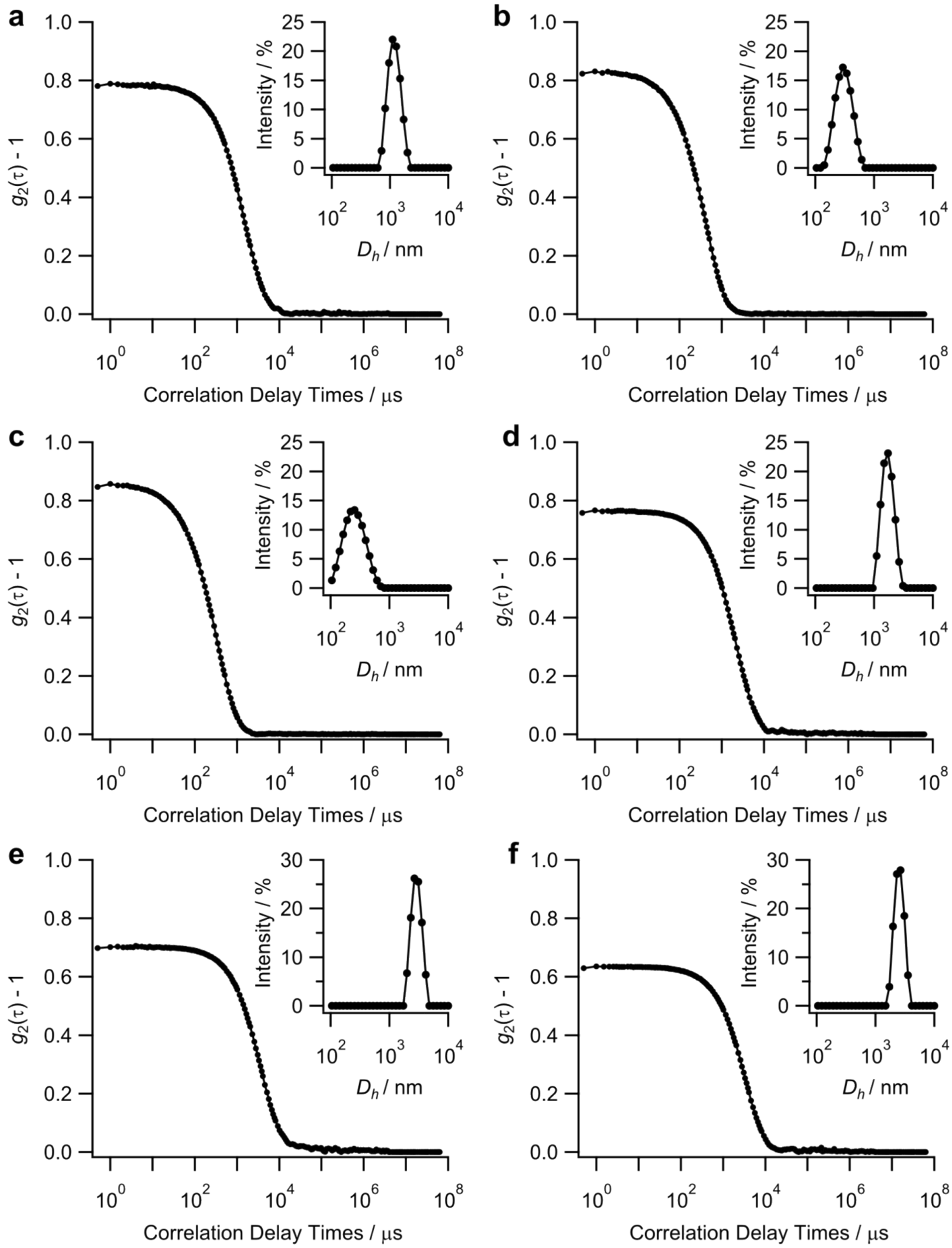

Figure S2. Dynamic light scattering data of prepared coacervates one minute after coacervation at PDDA (100 kDa, 20 mM)/FMOC-AA molar ratios of 1:1 (**a**), 1:0.85 (**b**), 1:0.5 (**c**), 0.85:1 (**d**) and 0.5:1 (**e**); and PDDA (8.5 kDa, 20 mM)/FMOC-AA molar ratio of 1:1 (**f**). Main plots show raw correlograms and corresponding particle size distributions by intensity in the top right. D_h is the hydrodynamic diameter.

PDDA (Mw/kDa)	Ratio (PDDA:FMOC-AA)	Hydrodynamic Diameter / nm	Polydispersity Index
100	1 : 1	1234 ± 210	0.16 ± 0.02
100	1 : 0.85	280 ± 17	0.15 ± 0.05
100	1 : 0.5	234 ± 13	0.23 ± 0.03
100	0.85 : 1	2193 ± 450	0.07 ± 0.06
100	0.5 : 1	3553 ± 828	0.11 ± 0.03
8.5	1 : 1	2514 ±288	0.19 ± 0.16

Table S1. Hydrodynamic diameters and polydispersity indices obtained from DLS data (**Figure S2**) after one minute of coacervation ([PDDA] = 20 mM).

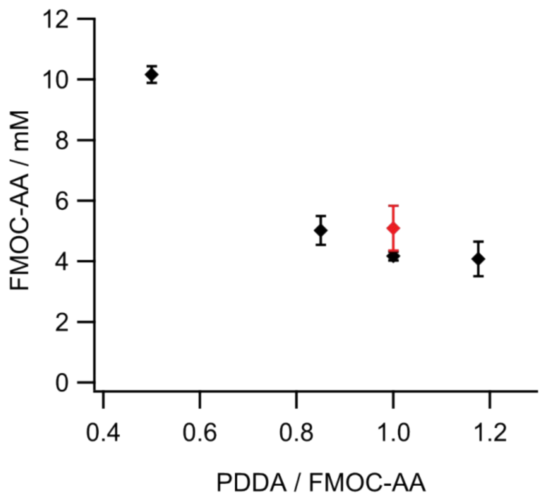

Figure S3. Plot of the concentration of FMOC-AA present in the continuous aqueous phase as a function of the total concentration of PDDA divided by the total concentration of FMOC-AA comprising the coacervate system prepared using 100 kDa (black) or 8.5 kDa (red) PDDA.

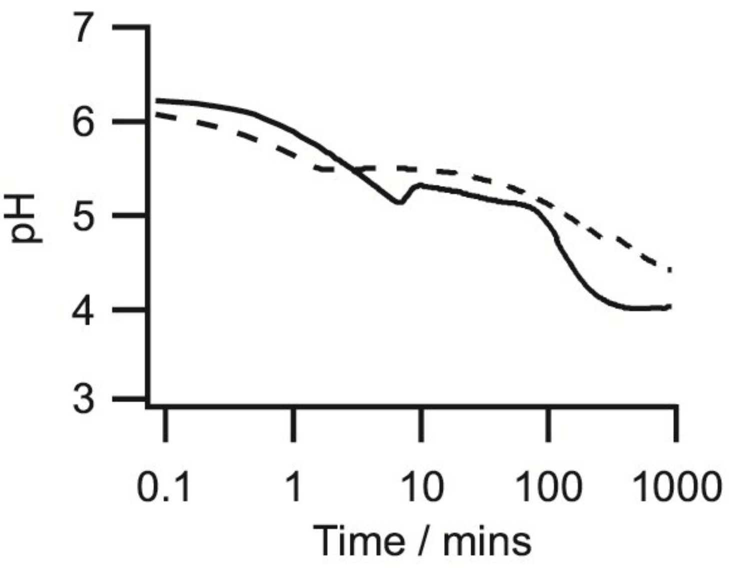

Figure S4. Plot showing change of pH with time after addition of 20 mM GDL to a PDDA/FMOC-AA coacervate (solid line) or a bulk solution containing 20 mM FMOC-AA (dashed line).

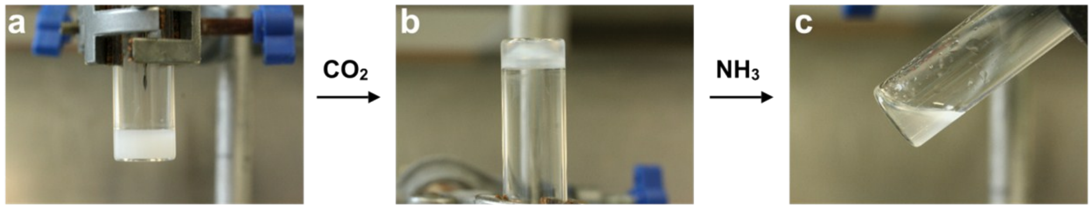

Figure S5. Photographic images of sample tubes demonstrating the reversible conversion of a PDDA (100 kDa)/FMOC-AA (1 : 1) system from a coacervate phase (**a**) to hydrogel phase (**b**) by addition of gaseous CO_2. Addition of gaseous NH_3 to the hydrogel re-establishes the coacervate phase (**c**). Formation of a self-standing hydrogel is shown by inversion of the sample tube and by the decrease in turbidity. Re-coacervation is accompanied by the formation of a turbid liquid dispersion.

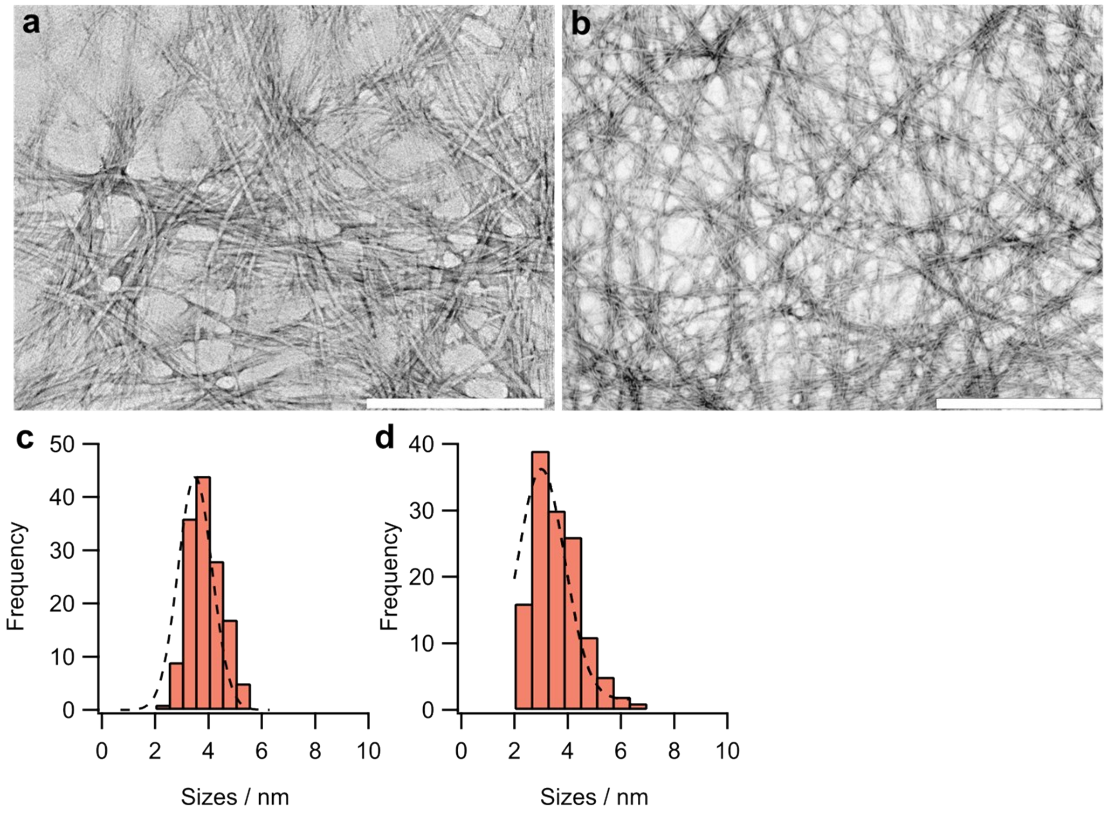

Figure S6. (**a**, **b**) TEM images of a 1:1 PDDA (100 kDa)/FMOC-AA hydrogel (**a**) (scale bar = 500 nm) and a 20 mM FMOC-AA hydrogel (**b**) (scale bar = 1000 nm). Fibres observed in micrographs are stained with uranyl acetate (1 wt % solution). (**c**,**d)**, Size distribution of FMOC-AA protofilament widths in 1:1 PDDA (100 kDa)/FMOC-AA hydrogel (**c**), and in control 20 mM FMOC-AA hydrogel (**d**).

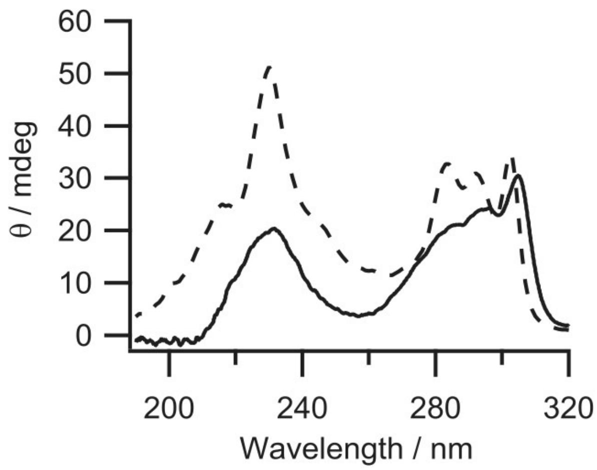

Figure S7. CD spectra for hydrogels produced by pH-mediated transformation in PDDA/FMOC-AA coacervate media (solid line) or bulk solutions of FMOC-AA (dashed line).

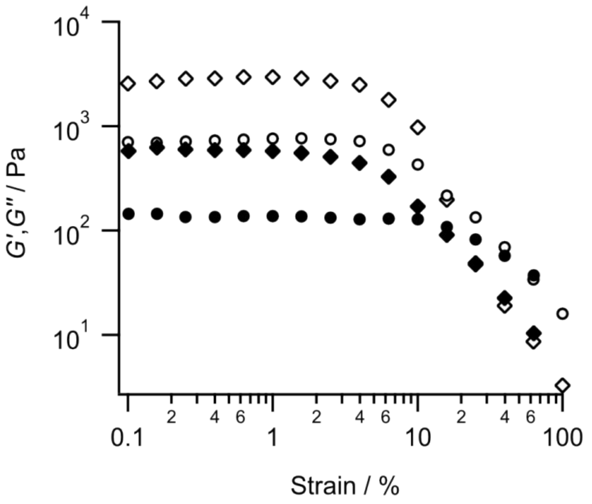

Figure S8. Oscillatory amplitude sweeps of a PDDA (100 kDa)/FMOC-AA 1 : 1 coacervate-derived peptide hydrogel (closed symbols) and a hydrogel prepared from 20 mM aqueous FMOC-AA hydrogel (open symbols). *G′* and *G″* are represented by diamonds and circles, respectively.

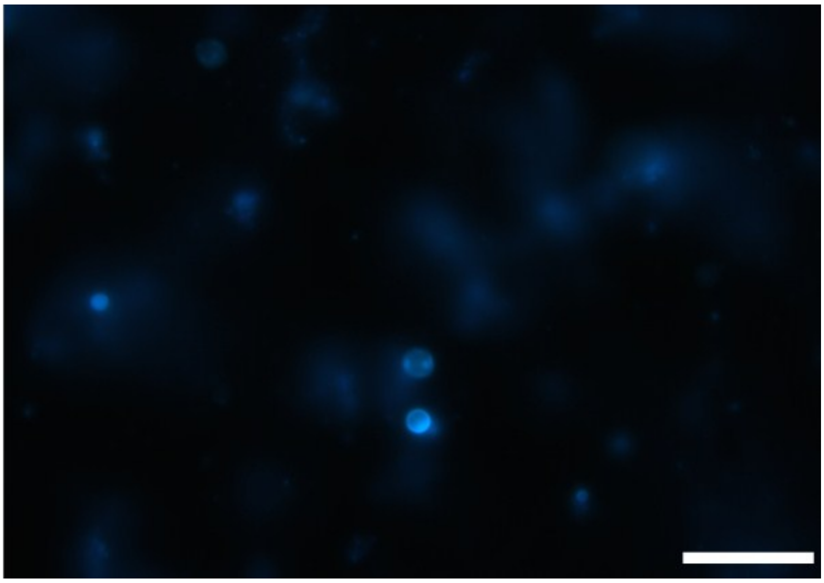

Figure S9. Epifluorescence microscopy image showing of kinetic aggregates formed on addition of 20 mM of HCl to PDDA (100 kDa)/FMOC-AA (1:1) coacervate micro-droplets; scale bar = 100 µm.

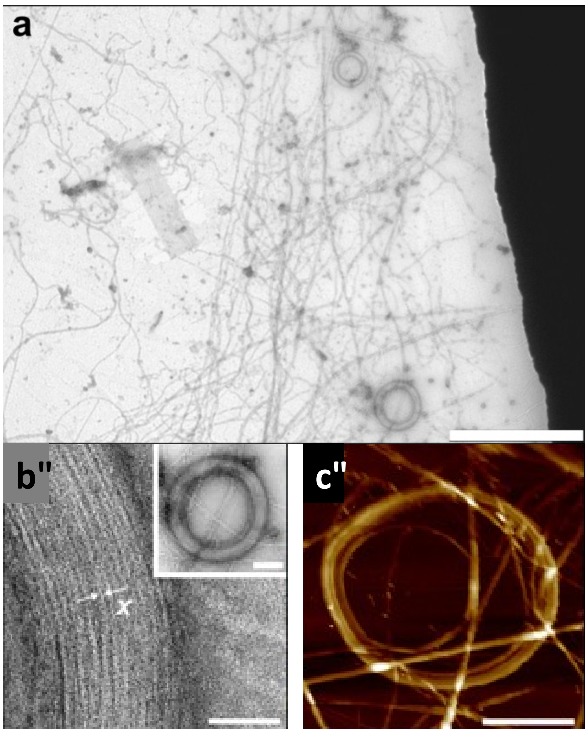

Figure S10. Formation of ring-like bundles of dipeptide nanofibres during transformation of PDDA/FMOC-AA coacervate micro-droplets into aster-shaped micro-architectures. (**a**) Low magnification TEM image showing two ring structures and fibrous network; scale bar = 2 µm. (**b**) high magnification TEM image of single ring-like structure (inset, scale bar = 200 nm) and high resolution image showing individual co-aligned dipeptide nanofilaments with an individual thickness of *ca*. 2 nm (scale bar = 50 nm. (**c**) AFM image of single ring-like bindle of co-aligned dipeptide nanofilaments; scale bar = 1 µm.

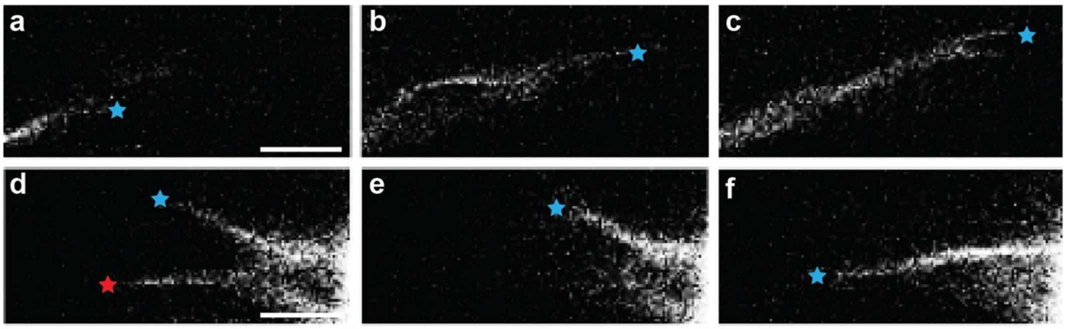

Figure S11. (**a-c**) Video images recorded every 5 s showing growth of an individual dipeptide nanofibre emanating from a PDDA/FMOC-AA coacervate micro-droplet. The tip of the growing filament is highlighted (blue star). Scale bar is 2 µm. (**d-f**) Video sequence showing two filaments (red and blue stars) growing and combining over time. Times between images: (**d**) and (**e**) = 5 s, (**e**) and (**f**) = 15 s. Scale bar is 2 µm.

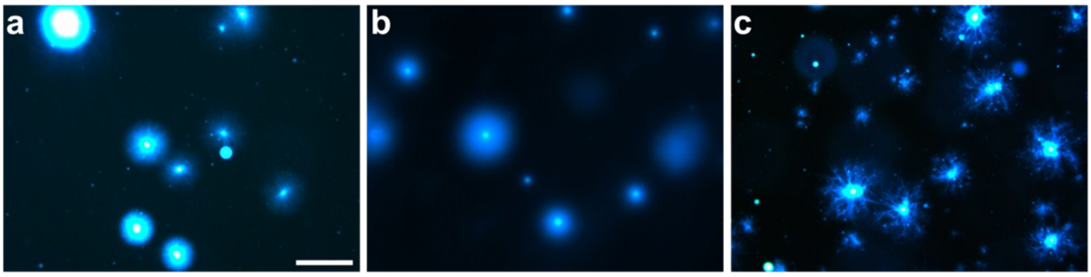

Figure S12. Fluorescence microscopy images showing GDL-mediated transformations of micro-droplets to aster-like microstructures for coacervates prepared from (**a**) PDDA (100 kDa)/FMOC-AA (1 : 0.85), (**b**) PDDA (100 kDa)/FMOC-AA (1 : 0.5), and (**c**) PDDA (8.5 kDa)/FMOC-AA (1 : 1). Scale bar = 100 µm for all images.

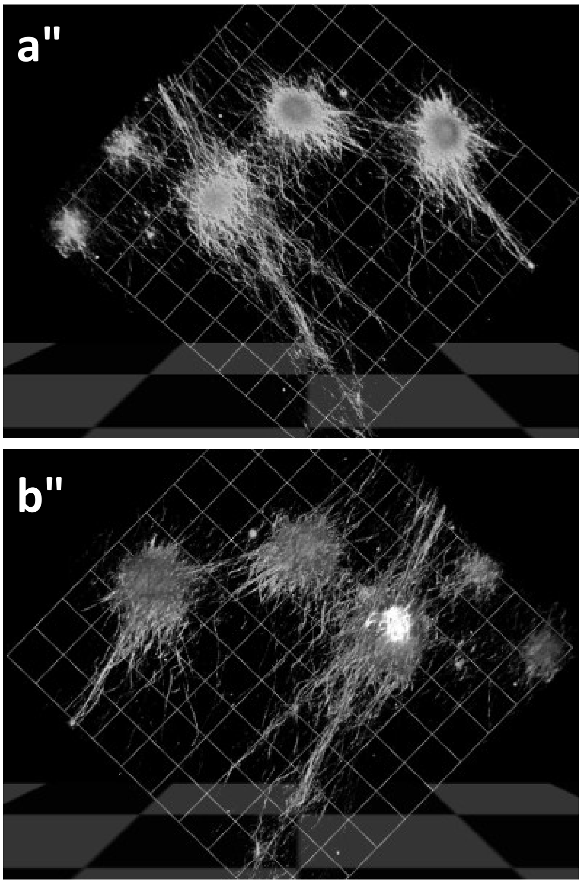

Figure S13. Confocal fluorescence 3D stacked images of (**a**) bottom-view and (**b**) top-view of a self-structuring dipeptide network showing fibres emanating from individual coacervate micro-droplets and spreading across the glass slide. Image was recorded *ca*. 40 mins after GDL addition; scale per grid square length = 18 µm. All samples were stained with Hoechst 33258 dye.

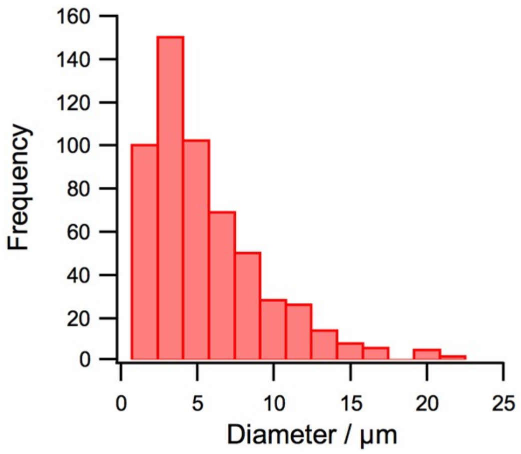

Figure S14. Size distribution profile for coacervate droplets produced by centrifugation and agitation.
